# P-1233. Less is More? A Retrospective Comparison of Twice and Thrice Daily Dosing of Metronidazole in Confirmed Anaerobic Infections

**DOI:** 10.1093/ofid/ofaf695.1425

**Published:** 2026-01-11

**Authors:** Hayley W Hamilton, Randi Silcox, Sasha Ward, Laura Comalander, Emily A Plauche

**Affiliations:** Ascension Sacred Heart Pensacola, Pensacola, Florida; Ascension Sacred Heart Pensacola, Pensacola, Florida; Ascension Sacred Heart Pensacola, Pensacola, Florida; Ascension Sacred Heart Pensacola, Pensacola, Florida; Ascension Sacred Heart Hospital, Pensacola, Florida

## Abstract

**Background:**

Metronidazole is commonly used for anaerobic infections due to its affordability, effectiveness, and tolerability. Its half-life is 6 to 10 hours with studies showing therapeutic serum levels at 12 hours. Studies comparing BID to TID dosing in presumed and confirmed anaerobic infections have shown similar clinical outcomes. Based on emerging evidence and its pharmacokinetics, our hospital system updated the standard dosing of metronidazole from 500mg TID to 500mg BID for most indications. The purpose of this study was to evaluate the effectiveness of this dosing in confirmed anaerobic infections.

**Figure:**
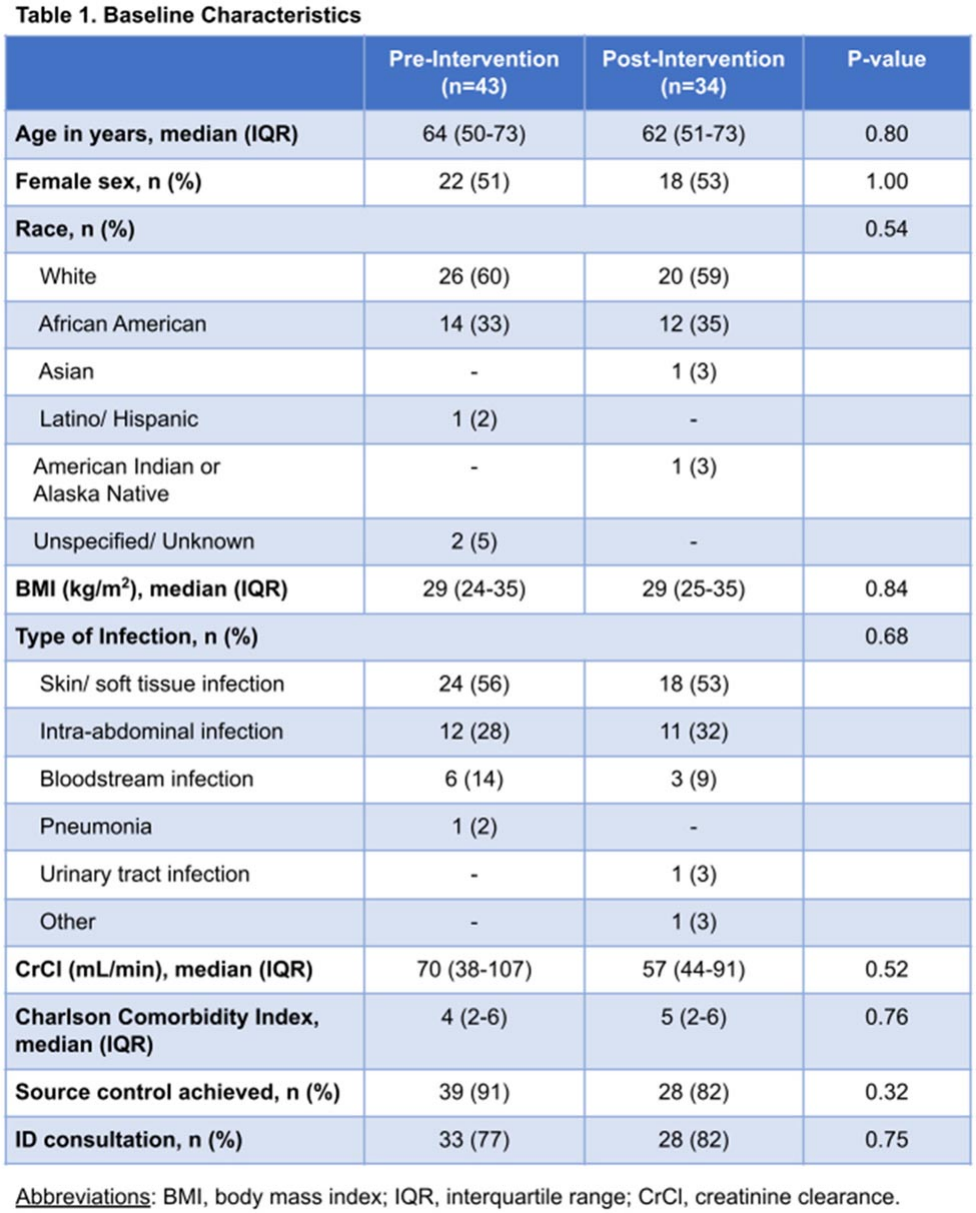

**Figure:**
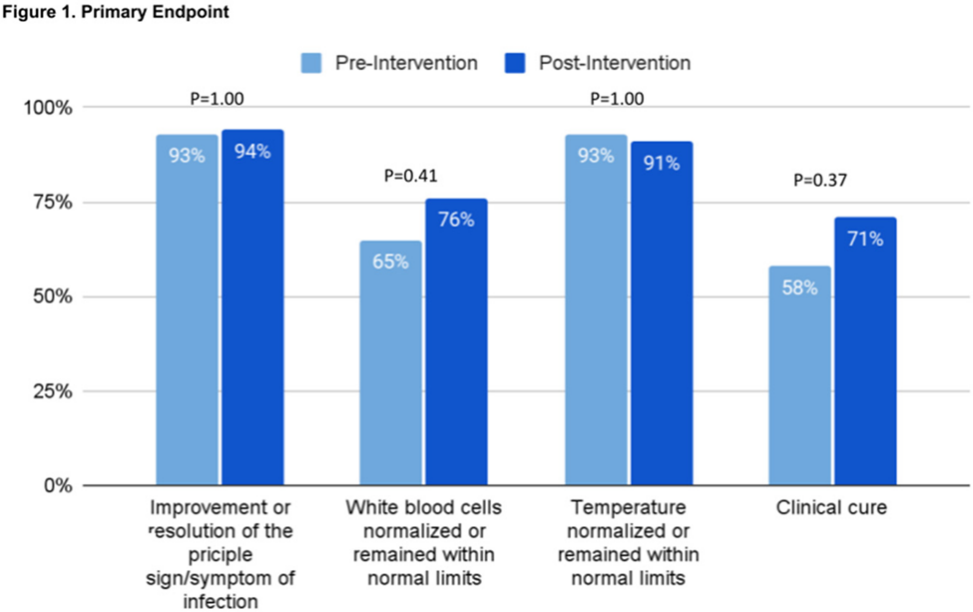

**Methods:**

This was an IRB exempt, retrospective, quasi-experimental study at two sites in a community teaching hospital system. In June 2023, the standard dosing of metronidazole was changed to BID. Pre-intervention data included patients who received TID dosing from January 2022 to April 2023. Post-intervention data included patients who received BID dosing from November 2023 to June 2024. Adult inpatients included in this study received metronidazole for at least 72 hours for a confirmed anaerobic infection. Patients were excluded if they had a central nervous system infection, *Clostridioides difficile* infection, parasitic or amoebic infection, *Helicobacter pylori* infection, or metronidazole for surgical prophylaxis.

The primary outcome was frequency of clinical cure (improvement or resolution of the principal sign or symptom of infection with normalization of white blood cells and temperature at the end of therapy or discharge). Secondary outcomes included inpatient mortality, length of stay, therapy escalation, readmission due to the same infection, and metronidazole spend.

**Figure:**
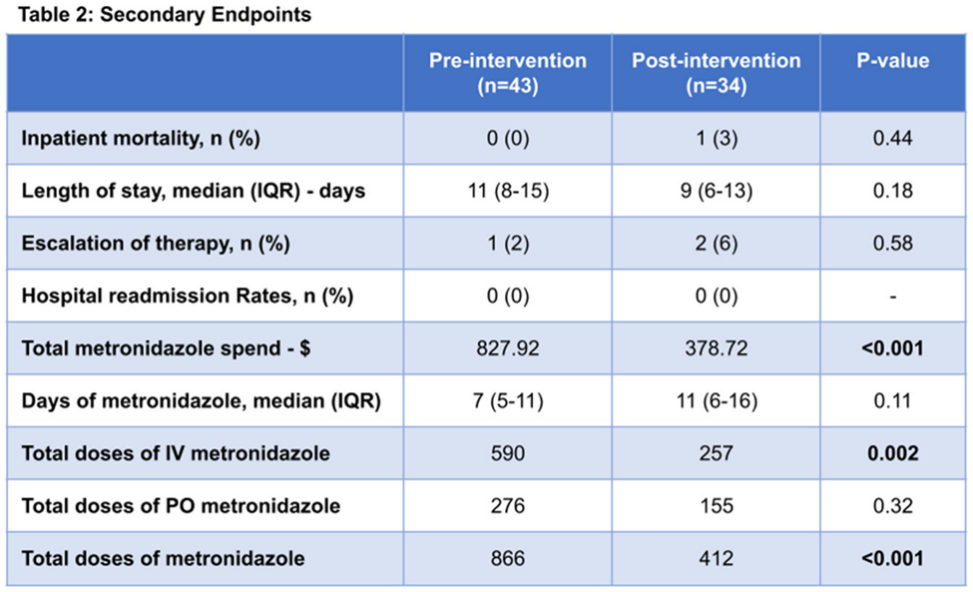

**Results:**

There were 77 patients included in the study (43 pre-intervention; 34 post-intervention). Baseline characteristics were similar (Table 1). Clinical cure occurred in 25 patients (58%) in the pre-intervention group and 24 patients (71%) in the post-intervention group (p=0.37) (Figure 1). Secondary outcomes are shown in Table 2.

**Conclusion:**

No significant differences in clinical outcomes were found between metronidazole BID and TID dosing for confirmed anaerobic infections. This study adds to existing data to support BID dosing of metronidazole.

**Disclosures:**

All Authors: No reported disclosures

